# ^1^H, ^15^N and ^13^C backbone and side-chain resonance assignments of Amblyomin-X Kunitz domain

**DOI:** 10.1007/s12104-026-10275-4

**Published:** 2026-08-24

**Authors:** Vitor S. Almeida, Virginnia C. Nogueira, Livia S. O. Conti, Ana Paula B. Costa, Ana Paula Valente

**Affiliations:** 1 Institute of Medical Biochemistry Leopoldo de Meis (IBqM), Federal University of Rio de Janeiro (UFRJ), Rio de Janeiro, RJ 21941‑590 Brazil; 2 National Center for Structural Biology and Bioimaging (CENABIO), Federal University of Rio de Janeiro (UFRJ), Rio de Janeiro, RJ 21941‑590 Brazil

**Keywords:** Anticoagulant, Amblyomin-X, Factor Xa, NMR assignments

## Abstract

Amblyomin-X is a Kunitz-type inhibitor of factor Xa (FXa) from the tick *Amblyomma sculptum* with promising anticoagulant activity and potential therapeutic applications. Despite the relevance, the molecular basis of its interaction with FXa remains poorly understood, limiting a detailed understanding of its mechanism of action. In this study, we report the ^1^H, ^13^C, and ^15^N resonance assignments of the Amblyomin-X Kunitz domain obtained by multidimensional NMR spectroscopy. High-quality spectra enabled extensive assignment, reaching 97.9% completeness for backbone assignments and 79.8% for side-chain assignments. The ^1^H-^15^N HSQC spectrum shows excellent signal dispersion, consistent with a well-folded protein in solution. Furthermore, chemical shift-based structure analysis reveals elements that agree with the canonical Kunitz fold, including characteristic β-strands and helical regions. These results provide the first detailed NMR characterization of the Amblyomin-X Kunitz domain l. The resonance assignments presented here constitute a critical foundation for future structural and dynamical studies, including analyses of protein-ligand interactions. Ultimately, this work contributes to a deeper understanding of the molecular determinants governing FXa inhibition by Amblyomin-X and supports ongoing efforts to develop novel anticoagulant strategies based on Kunitz-type inhibitors.

## Biological context

Pathophysiological conditions such as thrombosis and cancer are associated with dysregulation of the blood coagulation cascade, which is normally maintained through a finely tuned balance between procoagulant and anticoagulant pathways (Hook and Abrams 2012; Lima and Monteiro 2013). In prothrombotic states, the identification of novel anticoagulant agents has therefore become a key strategy for developing improved therapeutic interventions (Francischetti et al. 2002; Ma et al. 2013). In this context, our group recently determined the high-resolution structure and dynamics of Ixolaris, an exogenous tissue factor pathway inhibitor (TFPI) from the salivary glands of the tick *Ixodes scapularis*, and elucidated its noncanonical mechanism of factor X inhibition (De Paula et al. 2017; Paula et al. 2019).

In this context, Amblyomin-X - a Kunitz-type inhibitor of factor Xa (FXa) derived from the salivary glands of the tick *Amblyomma sculptum*, is a 108-residue protein containing seven cysteines. Six of these residues are located within a single Kunitz domain in the N-terminal region (residues 1–58), whereas the remaining cysteine residue resides in the intrinsically disordered C-terminal region (residues 59–108) (Batista et al. 2008, 2010). Despite its functional relevance, the molecular mechanism underlying the interaction between Amblyomin-X and FXa remains incompletely understood (Mesquita Pasqualoto et al. 2014).

Here, we report the ^1^H, ^13^C, and ^15^N resonance assignments for the backbone and side-chain atoms of the Amblyomin-X Kunitz domain, along with secondary-structure information derived from chemical shifts. These data provide a critical foundation for determining its solution structure and dynamics by NMR and for advancing our understanding of the molecular basis of FXa inhibition by Amblyomin-X.

## Methods and experiments

### Protein expression and purification

The Amblyomin-X Kunitz domain (corresponding to residues 1–63) was synthesized and inserted into a bacterial expression vector pET-32a at the 5′ NcoI and 3′ BamHI restriction sites by Genscript. ^13^C/^15^N labeled NMR samples were produced in *Escherichia coli* Rosetta-gami B (DE3) harboring the expression construct. The bacterial culture was grown in M9 minimal medium (Na_2_HPO_4_⋅12H_2_O 17 g/L, KH_2_PO_4_ 3 g/L, NaCl 0.5 g/L, MgSO_4_ 0.24 g/L, CaCl_2_⋅2H_2_O 14.7 g/L, thiamine 1 g/L)(De Paula et al. 2017) supplemented with [^13^C]-glucose (3 g/L) and/or ^15^NH_4_Cl (1 g/L), as the only sources for carbon and nitrogen, respectively. The M9 medium was also supplemented with Celtone^®^ Base Powder [U-^15^N,^13^C] 1 g/L (Cambridge Isotopes Laboratories), yeast nitrogen base without amino acids 1.7 g/L (SIGMA), and MEM Vitamin Solution (SIGMA). Ampicillin (50 µg/mL) and Chloramphenicol (34 µg/mL) were incorporated into the selective medium. The bacterial culture was incubated at 37 °C, stirred at 120 rpm until it reached an OD_600_ of 0.6. Protein expression was induced by the addition of 0.4 mM isopropyl-β-d-thiogalactopyranoside (IPTG) for 20 h, shaking at 120 rpm at 15°C.

The cells were harvested by centrifugation for 10 min at 6641×*g* at 4 °C and resuspended in lysis buffer [50 mM Tris–HCl (pH 7.6), 500 mM NaCl, 10 mM Imidazole, 10 µg/mL lysozyme, and 1 mM phenylmethanesulfonylfluoride (PMSF)] and disrupted by 10 cycles of sonication (60 W, 30 s *ON* e 60 s *OFF*). The bacterial lysate was clarified by centrifugation at 14,942×*g* for 30 min at 4 °C. Soluble protein in the supernatant of cell lysate was purified by a Ni^2+^-affinity chromatography step using a His-Trap HP 5 mL column (GE Healthcare) previously equilibrated in 50 mM Tris–HCl buffer (pH 7.6), 500 mM NaCl, 10 mM imidazole. TRX-His_6_-Amblyomin-X Kunitz domain was eluted in 50 mM Tris–HCl buffer (pH 7.6), 500 mM NaCl, 200 mM imidazole, and subsequently dialyzed against 50 mM Tris–HCl buffer (pH 7.6), 100 mM NaCl. The removal of the N-terminal TRX-His_6_ tag was achieved using 5U of Enterokinase (EKMax™). Amblyomin-X Kunitz domain was further purified to homogeneity by reversed-phase chromatography using a PRP-3 column (Hamilton, 7.0 mm × 305 mm) with a linear acetonitrile gradient (10–90%) containing 0.1% TFA. The resulting sample was freeze-dried under vacuum. SDS–PAGE revealed protein purity of > 98%. Intact Amblyomin-X Kunitz domain mass measurements (7254.01 Da) were performed on a NanoAcquity UPLC system (Waters) interfaced with a SYNAPT G1 HDMS high-resolution mass spectrometer (Waters), operated with a nano-electrospray ionization (nano-ESI) source.

### NMR experiments

Samples containing 0.25 mM Amblyomin-X Kunitz domain in 20 mM sodium phosphate buffer (pH 6.0), 2 mM azide, 10% D_2_O were used for NMR measurements. All NMR experiments were carried out at 308 K on a Bruker Avance III HD 800 MHz equipped with a TXI 5 mm triple-resonance probe. Sequential backbone resonance assignments were achieved through analysis of the multidimensional heteronuclear experiments: 2D ^1^-^15^ N HSQC, 2D ^1^-^13^ C HSQC, 3D HNCACB, 3D CBCA(CO)NH, 3D HNCO (Ikura et al. 1990; Wittekind and Mueller 1993). The aliphatic side-chain assignment was obtained using HBHA(CO)NH (Grzesiek and Bax 1993), hCCH-TOCSY, HcCH-TOCSY (Kay et al. 1993), ^15^N-edited NOESY-HSQC, and ^13^C-edited NOESY-HSQC (Sattler et al. 1999), optimized for both aliphatic and aromatic region detection experiments, and with the help of complementary 3D (H)CC(CO)NH (Logan et al. 1992). All triple-resonance experiments were performed using non-uniform sampling at 15%, except for NOESY experiments, which used 50% sampling. 2D ¹H–¹⁵N HSQC spectra of the Amblyomin-X Kunitz domain were acquired at different temperatures (278 K, 308 K, and 318 K) on a Bruker Avance III 900 MHz spectrometer equipped with a TXI 5 mm triple-resonance probe. All NMR data were processed using NMRPipe (Delaglio et al. 1995), and the resonances were analyzed using the software CCPNMR Analysis v.3 (Skinner et al. 2016), both of which are available on the NMRbox platform (Maciejewski et al. 2017).

### Assignment and data deposition

**Fig. 1:**
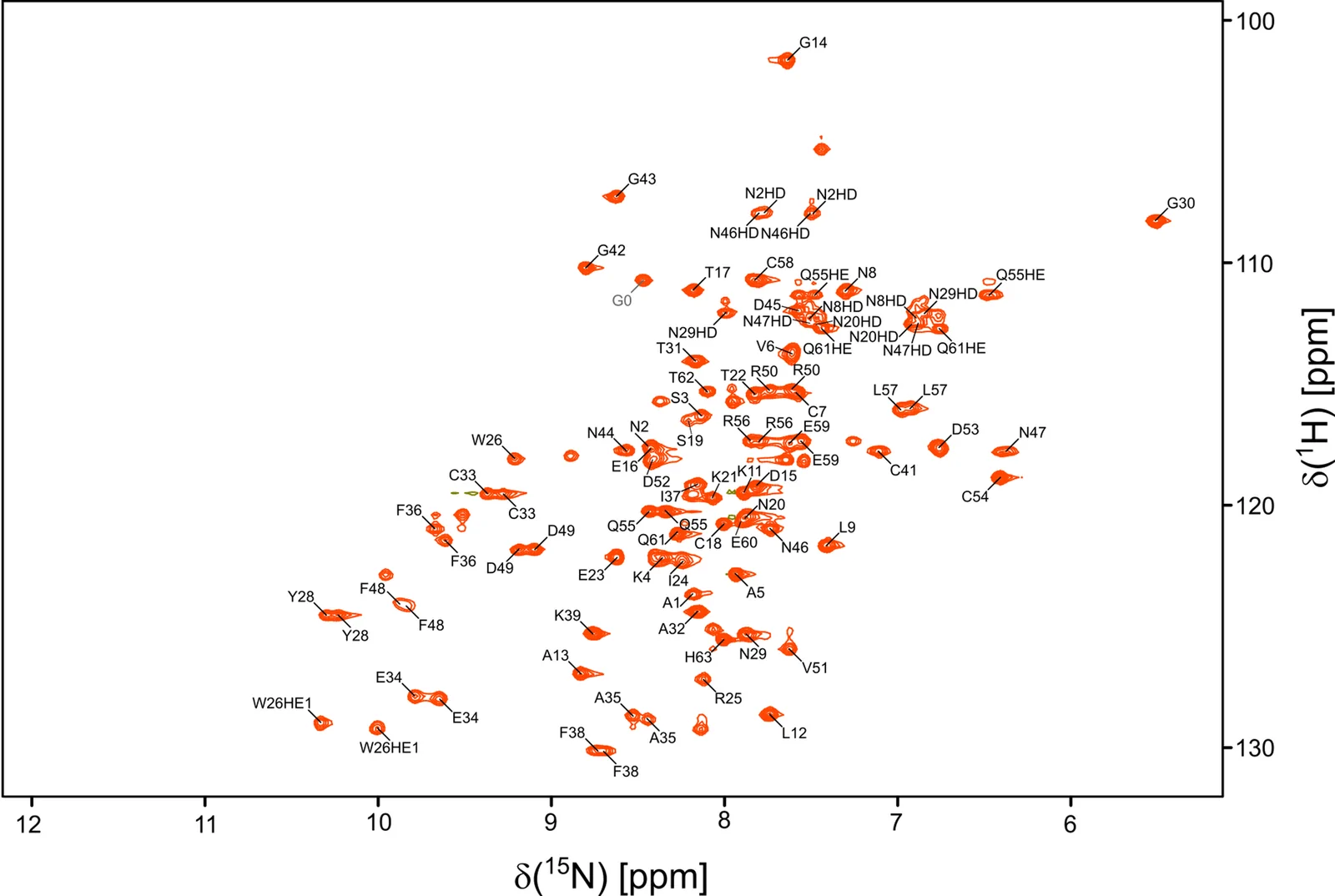
2D ^1H^-^15^N HSQC spectrum of Amblyomin-X Kunitz Domain. The spectrum was recorded at 308 K on a Bruker Avance III 800 MHz spectrometer using a 0.2 mM protein sample in 20 mM phosphate buffer (pH 6.0), 2 mM azide, and 10% D_2_O. The spectrum contains unassigned peaks of unknown origin

The ^1^H-^15^N HSQC spectrum of Amblyomin-X Kunitz Domain at 308 K exhibits well-dispersed NMR resonances and good resolution, indicating a folded protein structure (Fig. 1). A total of 97.9% of the backbone nuclei (100% ^13^Cα, 100% ^13^Cβ, 95.24% ^13^C′, 95.24% ^15^N, 100% H_α_, 96.77% amide HN) were assigned, while assignment of the side chain is 79.8% complete. The chemical shifts have been deposited in the BioMagResBank (http://bmrb.wis.edu)under Accession Number 53,378.

**Fig. 2:**
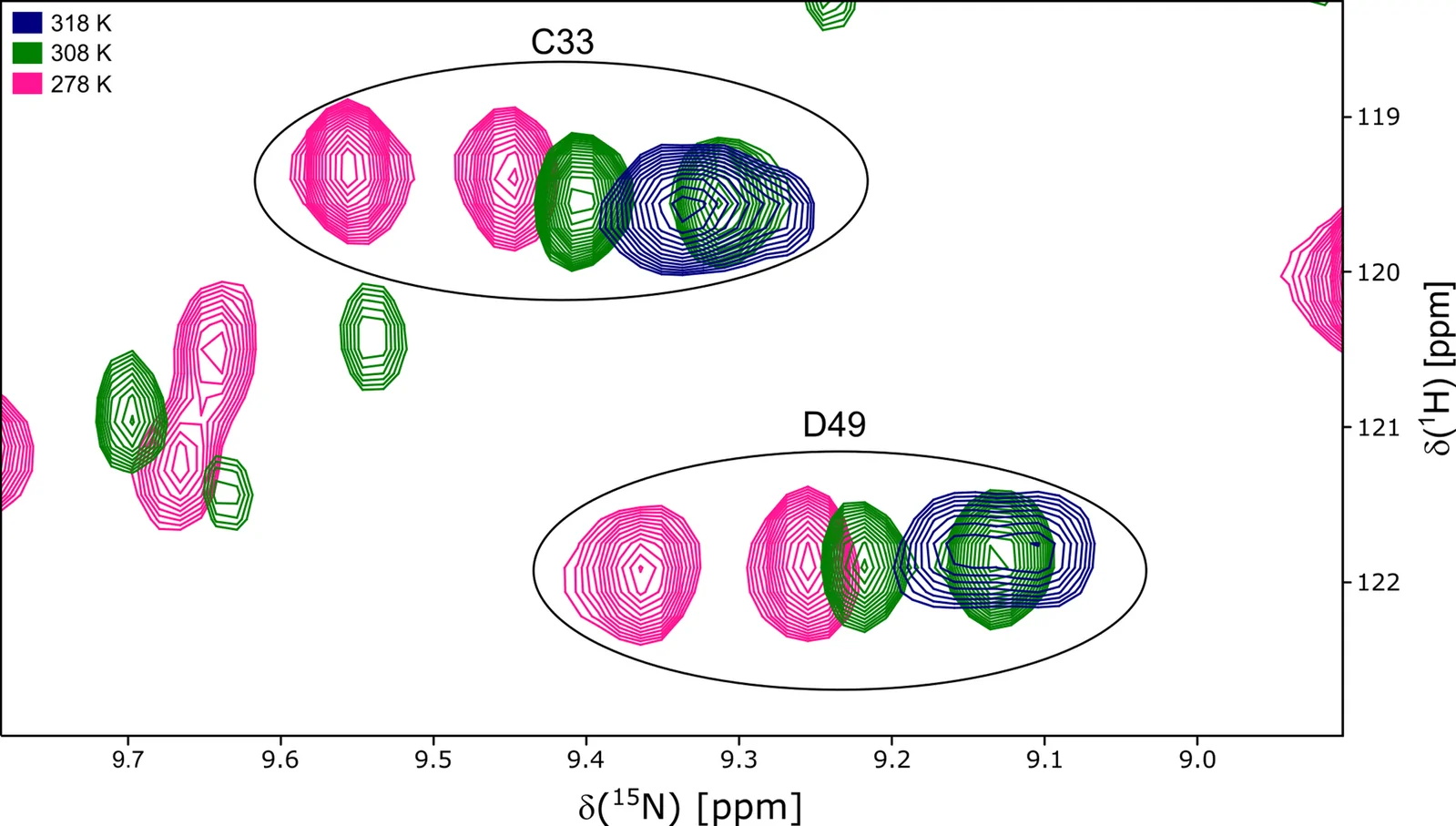
Zoomed region of the 2D ^1^H-^15^ N HSQC spectra of the Amblyomin-X Kunitz domain showing the C33 and D49 resonances. Spectra were acquired at 278, 308, and 318 K (pink, green, and blue, respectively) on a Bruker Avance III 900 MHz spectrometer using a 0.2 mM protein sample in 20 mM phosphate buffer (pH 6.0) containing 2 mM sodium azide and 10% D₂O

The ^1^H-^15^N HSQC spectrum exhibits additional peaks (indicated by the residue number) corresponding to duplicated spin systems for residues W26, Y28, C33, E34, A35, F36, F38, F48, D49, R50, Q55, R56, L57, and E59. For the resonance assignment, the more intense peak in each pair was selected. These duplicated resonances are observed for residues located near the Cys33–Cys54 disulfide bond and likely reflect conformational heterogeneity arising from disulfide-bond isomerization. Notably, increasing the experimental temperature resulted in temperature-dependent chemical-shift changes in several duplicated resonances, which progressively moved toward coalescence (Fig. 2). This behavior is consistent with a conformational exchange process involving interconversion between two conformational states, similar to that reported for BPTI (Otting et al. 1993). Further studies are underway to characterize this exchange process in greater detail and to determine its associated timescale.

The spectrum contains several unassigned resonances that likely originate from the residual amino acids (AMG) remaining after enterokinase cleavage of the expression construct, for which only a Gly could be assigned (labeled G_0_). Additional unassigned peaks are of unknown origin and may correspond to residues that undergo substantially larger chemical shift changes between the two conformational states, preventing them from appearing as readily identifiable resonance pairs in the spectrum. These resonances could not be assigned because the triple-resonance experiments did not provide sufficient information for unambiguous assignments. Importantly, no evidence was found for isomerization involving any other disulfide bond.

Importantly, the observed spectral heterogeneity is unlikely to arise from the presence of distinct protein species. First, the duplicated spin systems exhibit essentially identical Cα and Cβ chemical shifts, and therefore did not hamper the triple-resonance assignment strategy. Furthermore, under both reducing and non-reducing conditions, the protein migrated as a single band at the expected molecular weight on SDS-PAGE. In addition, MALDI-TOF mass spectrometry confirmed the expected molecular mass (7314.82 Da), providing no evidence for alternative species, disulfide-mispaired forms, or oligomeric assemblies.

### Secondary structure information from the ^13^C chemical shifts

**Fig. 3:**
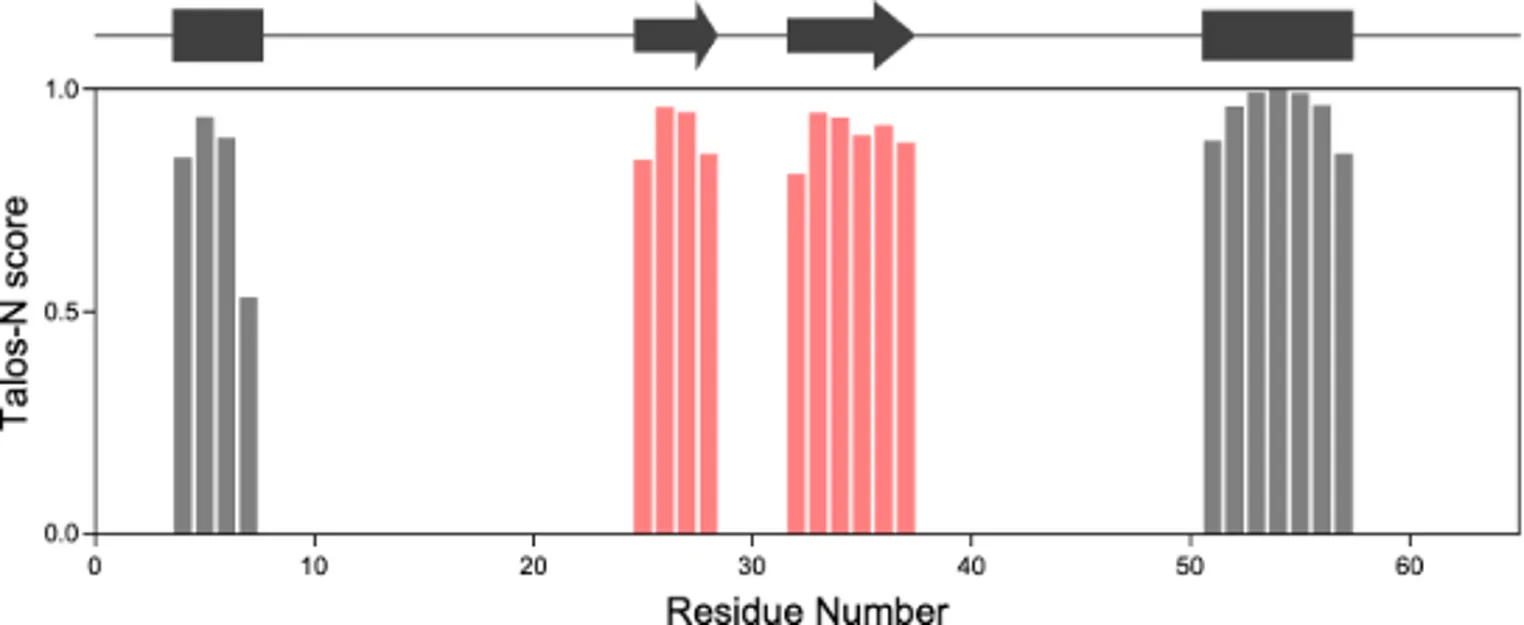
Secondary structure elements of the Amblyomin-X Kunitz Domain predicted by Talos-N. The Talos-N index is plotted as a function of the Amblyomin-X Kunitz Domain residues. The locations of predicted α-helices (grey) and β-strands (red) are indicated by rectangles and arrows, respectively

Amblyomin-X Kunitz domain secondary-structure elements (Fig. 3) were predicted from chemical shifts using Talos-N (Shen and Bax 2013). According to Talos-N prediction, the Amblyomin-X Kunitz domain presents two α-helices (residues 4–7 and 51–57) and two β-strands (residues 25–28 and 32–37), which are connected by long loops (residues 8–24, 38–50). In summary, the chemical shift assignments reported here will be important for further determining the structure and dynamics of the Amblyomin-X Kunitz domain in solution by NMR.

## Data Availability

Publicly available in a repository: Chemical shift assignments for the Amblyomin-X Kunitz domain reported in this manuscript are deposited in the Biological Magnetic Resonance Bank (BMRB) under access ID 53378.
